# Airway *Pseudomonas aeruginosa* density in mechanically ventilated patients: clinical impact and relation to therapeutic efficacy of antibiotics

**DOI:** 10.1186/s13054-021-03488-7

**Published:** 2021-02-11

**Authors:** Yohei Migiyama, Shinya Sakata, Shinji Iyama, Kentaro Tokunaga, Koichi Saruwatari, Yusuke Tomita, Sho Saeki, Shinichiro Okamoto, Hidenori Ichiyasu, Takuro Sakagami

**Affiliations:** 1 Department of Respiratory Medicine, Kumamoto University Hospital, Kumamoto, Japan; 2 Demachi Naika, Kumamoto, Japan; 3 Department of Critical Care Medicine, Kumamoto University Hospital, Kumamoto, Japan

**Keywords:** Airway bacterial density, Mechanical ventilation, *Pseudomonas aeruginosa*, Ventilator-associated lower respiratory tract infection

## Abstract

**Background:**

The bacterial density of *Pseudomonas aeruginosa* is closely related to its pathogenicity. We evaluated the effect of airway *P. aeruginosa* density on the clinical course of mechanically ventilated patients and the therapeutic efficacy of antibiotics.

**Methods:**

We retrospectively analyzed data of mechanically ventilated ICU patients with *P. aeruginosa* isolated from endotracheal aspirates. Patients were divided into three groups according to the peak *P. aeruginosa* density during ICU stay: low (≤ 10^4^ cfu/mL), moderate (10^5^‒10^6^ cfu/mL), and high (≥ 10^7^ cfu/mL) peak density groups. The relationship between peak *P. aeruginosa* density and weaning from mechanical ventilation, risk factors for isolation of high peak density of *P. aeruginosa*, and antibiotic efficacy were investigated using multivariate and propensity score-matched analyses.

**Results:**

Four-hundred-and-sixty-one patients were enrolled. Patients with high peak density of *P. aeruginosa* had higher inflammation and developed more severe respiratory infections. High peak density of *P. aeruginosa* was independently associated with few ventilator-free days on day 28 (*P* < 0.01) and increased ICU mortality (*P* = 0.047). Risk factors for high peak density of *P. aeruginosa* were prolonged mechanical ventilation (odd ratio [OR] 3.07 95% confidence interval [CI] 1.35‒6.97), non-antipseudomonal cephalosporins (OR 2.17, 95% CI 1.35‒3.49), hyperglycemia (OR 2.01, 95% CI 1.26‒3.22) during ICU stay, and respiratory diseases (OR 1.9, 95% CI 1.12‒3.23). Isolation of commensal colonizer was associated with lower risks of high peak density of *P. aeruginosa* (OR 0.43, 95% CI 0.26‒0.73). Propensity score-matched analysis revealed that antibiotic therapy for patients with ventilator-associated tracheobronchitis improved weaning from mechanical ventilation only in the high peak *P. aeruginosa* group.

**Conclusions:**

Patients with high peak density of *P. aeruginosa* had worse ventilator outcome and ICU mortality. In patients with ventilator-associated tracheobronchitis, antibiotic therapy was associated with favorable ventilator weaning only in the high peak *P. aeruginosa* density group, and bacterial density could be a good therapeutic indicator for ventilator-associated tracheobronchitis due to *P. aeruginosa*.
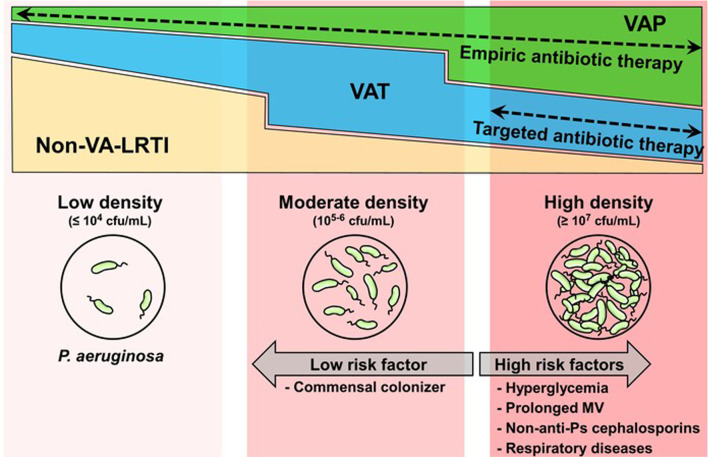

## Background

*Pseudomonas aeruginosa* (PA) is the most prevalent gram-negative pathogen in intensive care units (ICUs) and is the leading cause of respiratory infections among patients receiving mechanical ventilation (MV). Ventilator-associated lower respiratory tract infection (VA-LRTI) comprises ventilator-associated tracheobronchitis (VAT) and ventilator-associated pneumonia (VAP). Because VAT represents a continuum of colonization and VAP, with overlap between the two conditions, antibiotic therapy in patients with VAT remains controversial [1–3]. Furthermore, the increasing emergence of multidrug-resistant PA has become a global problem [4]. Therefore, appropriate antibiotic stewardship for VA-LRTI due to PA is an important issue. PA frequently colonizes the airways and forms biofilms, which are factors in the development of VAP [5, 6]. An important role in these mechanisms is the bacterial communication system called quorum sensing. Using this system, PA can drastically change its virulence in response to the surrounding bacterial population, and quorum sensing-regulated virulence factors are expressed under conditions of high, but not low, bacterial density [7]. Quorum sensing plays an important role in the pathogenicity of PA, and its inhibitors have been reported to inhibit biofilm formation in vitro and improve survival during lung infections in animal models [8, 9]. Thus, bacterial density has a strong influence on the pathogenicity of PA. However, only a few small studies have examined the relationship between PA density and clinical features of patients undergoing MV, showing patients with high PA density were likely to have more virulent strains [10]. The present study aimed to investigate the effect of PA density on the clinical course and therapeutic efficacy of antibiotics in MV patients. We also evaluated the risk factors for a high PA density.

## Methods

### Study design and population

This was a retrospective cohort study performed at Kumamoto University Hospital, a tertiary care teaching hospital in Japan. We included all ICU patients with PA isolated from respiratory specimens during their ICU stay between January 2004 and December 2019. Patients younger than 15 years or requiring MV for less than 24 h were excluded from the study.

### Data collection and definitions

**Fig. 1:**
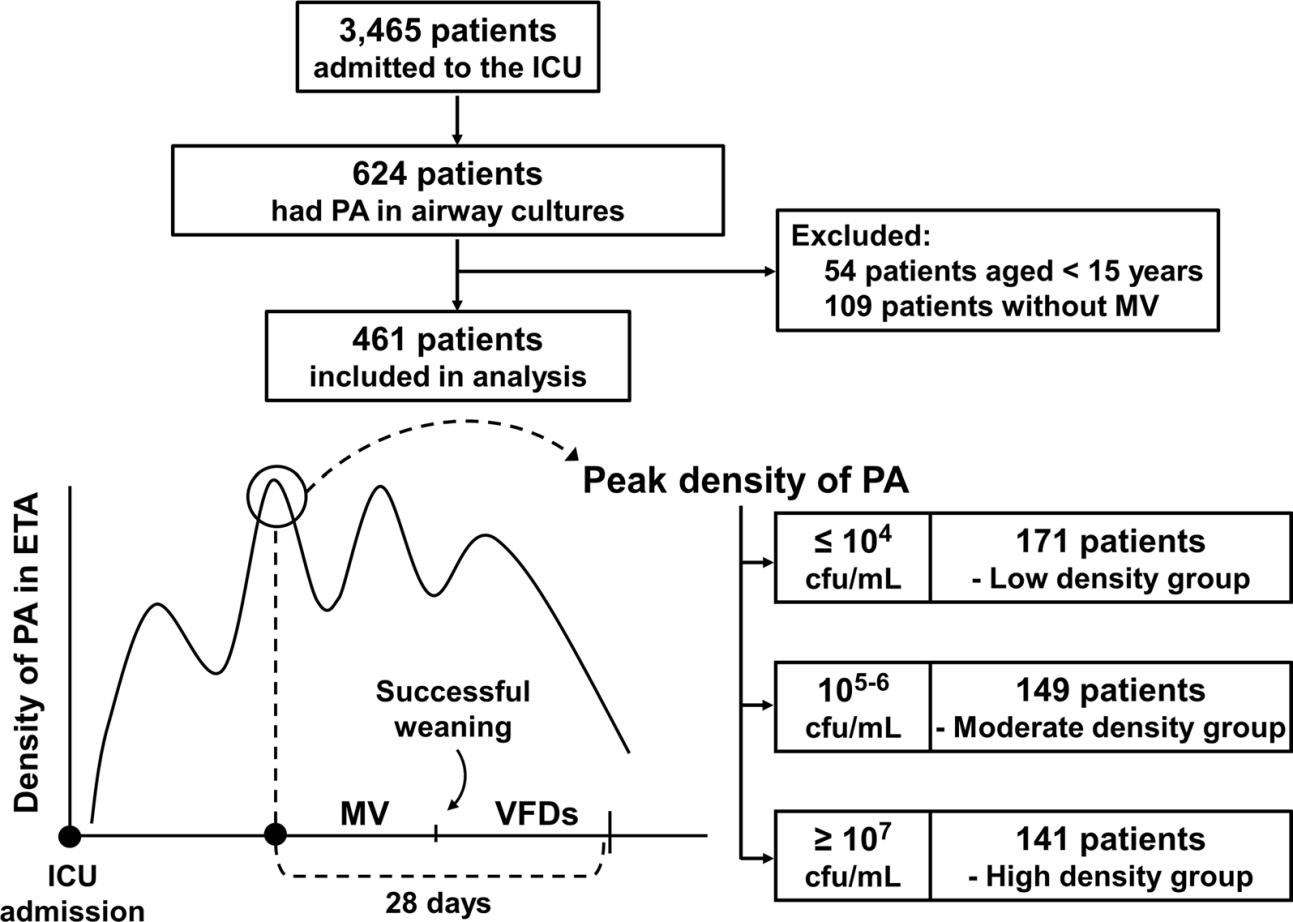
Patient flow diagram and definition in analysis. *ETA* endotracheal aspirates, *ICU* intensive care unit, *MV* mechanical ventilation, *PA Pseudomonas aeruginosa*, *VFDs* ventilator-free days

The patients were divided into three groups according to the peak density of PA in endotracheal aspirates (ETA) obtained during their ICU stay: low PA density (low PA; ≤ 10^4^ colony-forming units [cfu]/mL), moderate PA density (moderate PA; 10^5‒^10^6^ cfu/mL), and high PA density (high PA; ≥ 10^7^ cfu/mL) groups (Fig. 1). These cutoff points for the three groups were chosen as a bacterial density of 10^5^‒10^6^ cfu/mL in ETA is frequently used as the threshold for diagnosing VA-LRTI [1, 11, 12]. ETA samples were taken on admission and then once weekly during ICU stay. Additional cultures were performed when respiratory infection was suspected. For patients in whom the same peak density was detected multiple times, the first event was included in the analysis.

The diagnostic criteria for VA-LRTI included the following: at least one finding of fever (> 38 °C), hypothermia (< 36 °C), leukocytosis (> 12,000/μL), or leukopenia (< 4000/μL), in addition to purulent tracheal secretions. Patients who met the VA-LRTI criteria and had no new or progressive infiltrate on chest X-ray were diagnosed with VAT, whereas those with these chest X-ray findings were diagnosed with VAP [1, 13].

Disease severity on ICU admission was assessed using Acute Physiology and Chronic Health Evaluation (APACHE) II [14] and Sequential Organ Failure Assessment (SOFA) [15] scores. The simplified Clinical Pulmonary Infection Score (CPIS) [16] and SOFA score were also evaluated when the peak density of PA was detected. Patients' comorbid conditions were assessed using the Charlson comorbidity index [17]. Patients were considered to have hyperglycemia if blood glucose exceeded 200 mg/dL on two or more occasions during the ICU stay. To examine the risk factors for isolation of high PA in MV patients, we obtained data on the length of MV until the day when the peak density of PA was detected.

In the microbiological analysis of ETA, serial dilutions of samples were plated on blood, MacConkey, and chocolate agar. The numbers of colonies were counted after incubation, and pathogens were identified by an automated VITEK system (bioMérieux, Tokyo, Japan). Antibiotic sensitivities were determined using microdilution methods [18]. Samples were also evaluated using high-power field (HPF; 1,000 ×) optical microscopy after Gram staining for the semiquantitative measurement of neutrophils (0: none; 1 + : < 1 cell per 5 HPFs; 2 + : 1 cell per 5 HPFs; 3 + : 1 cell per HPF; and 4 + : ≥ 2 cells per HPF). The quality of the ETA samples could not be evaluated. A commensal colonizer was considered present if at least one of the following microorganisms was isolated: α-hemolytic streptococci, coagulase-negative staphylococci, non-pathogenic *Neisseria* sp., *Corynebacterium* sp., and *Candida* sp. [19–21]. Antibiotic therapy was defined as appropriate when PA showed in vitro susceptibility to empiric antibiotics (administered within 24 h of sampling for culture) for patients with VAP or targeted antibiotics (started or changed based on culture results) for patients with VAT [1, 22].

Clinical outcomes of this study were ventilator-free days (VFDs) on day 28, successful weaning rates, and ICU mortality. VFDs at day 28 were defined as the number of days that patients were both alive and free from MV during the 28 days after the peak density of PA was isolated (Fig. 1).

### Statistical analysis

Differences among the three groups were evaluated using the Kruskal‒Wallis test for continuous variables and the Chi-square test for categorical variables.

The impact of airway PA density on outcomes was investigated by multivariate linear regression analysis for VFDs at day 28, and by logistic regression analysis for ICU mortality, after adjusting for baseline patient characteristics. Multivariate logistic regression analysis was also used to identify risk factors for high PA in MV patients. All variables marginally significant in the univariate analysis (*P* < 0.15) and those variables associated with colonization or VAP due to PA (age, respiratory disease, APACHE II score at ICU admission, hyperglycemia during ICU stay, presence of commensal colonizers or other pathogenic bacteria in the airway, antibiotic therapy during ICU stay) were included in a logistic regression model, based on previous research [23–26].

Propensity score-matched analysis was performed to investigate the relationship between antibiotic therapy and outcomes. The propensity score was estimated using logistic regression analysis with the following 10 variables: age, sex, admission category, comorbid respiratory diseases, APACHE II score at ICU admission, SOFA, CPIS, albumin level, chest X-ray findings, and length of MV when peak density of PA was isolated. These variables were selected based on previous reports and clinical significance, suggesting a potential relationship with both antibiotic therapy assignment and outcomes [16, 27–29]. Patients who received appropriate antibiotic therapy (AAT) were matched 1:1 with those who did not, by using the nearest-neighbor matching method with a caliper of 0.2. After matching, clinical outcomes were compared with the Wilcoxon signed-rank test and Fisher’s exact test. Kaplan‒Meier analysis was also used to compare the time to successful weaning from MV. This propensity score-matched analysis was performed separately for each PA density group.

A *P* value < 0.05 was considered to be statistically significant. All statistical analyses were performed using SPSS Statistics 23.0 (SPSS Inc., Chicago, USA).

## Results

### Baseline characteristics of the study population

Of the 3465 screened patients, 461 patients were enrolled for analysis and classified into three group as follows: low PA (*n* = 171), moderate PA (*n* = 149), and high PA (*n* = 141) groups (Fig. 1). The baseline characteristics are shown in Table 1. No significant differences were observed among the three groups in terms of demographic characteristics, admission category, and illness severity parameters. Almost half of the patients were admitted to the ICU for reasons such as respiratory failure, pneumonia, and septic shock. The most common comorbid condition was malignancy. Patients in the high-PA group were more likely to have respiratory disease (*P* < 0.01). The details of respiratory disease were as follows: chronic obstructive pulmonary disease, 42%; interstitial lung disease, 31.8%; bronchial asthma, 21.6%; and bronchiectasis, 12.5%. Laboratory findings at ICU admission showed that patients in the high-PA group had significantly higher mean blood glucose levels and more hyperglycemia than other groups (*P* < 0.01).

**Table 1:** Patient’s characteristics at ICU admission

	Low-PA group (*n* = 171)	Moderate-PA group (*n* = 149)	High-PA group (*n* = 141)	*P* value
Age, median (IQR), years	66 (57‒74)	66 (56‒76)	67 (56‒77)	0.81
Sex, female	58 (33.9%)	50 (33.6%)	54 (38.3%)	0.64
Admission category				0.15
Medical	86 (50.3%)	82 (55%)	83 (58.9%)	
Elective surgery	52 (30.4%)	30 (20.1%)	34 (24.1%)	
Emergency Surgery	33 (19.3%)	37 (24.8%)	24 (17%)	
Main reason for ICU admission				
Respiratory failure	47 (27.5%)	35 (23.5%)	35 (24.8%)	0.7
Pneumonia	28 (16.4%)	17 (11.4%)	21 (14.9%)	0.44
Septic shock	22 (12.9%)	15 (10.1%)	19 (13.5%)	0.63
Neurological failure	14 (8.2%)	17 (11.4%)	12 (8.5%)	0.57
Severity parameter, median (IQR)				
APACHE II	22 (17‒28)	22 (18‒28)	22 (19‒28)	0.57
SOFA	9 (6‒12)	10 (7‒12)	9 (6‒12)	0.27
Main comorbidities				
Malignant disease	62 (36.3%)	53 (35.6%)	59 (41.8%)	0.48
Cardiovascular disease	58 (33.9%)	47 (31.5%)	43 (30.1%)	0.8
Neurologic disease	54 (31.6%)	39 (26.2%)	35 (24.8%)	0.36
Diabetes mellitus	36 (21.1%)	26 (17.5%)	31 (22%)	0.59
Respiratory disease	37 (21.6%)	17 (11.4%)	34 (24.1%)	< 0.01
Immunosuppression	33 (19.3%)	20 (13.4%)	30 (21.3%)	0.19
Charlson comorbidity index, median (IQR)	6 (4‒8)	6 (4‒7)	6 (4‒7)	0.72
Events prior to ICU admission				
Prior colonization or infection with PA	39 (22.8%)	25 (16.9%)	30 (21.3%)	0.41
Surgery within 30 days	91 (53.2%)	83 (55.7%)	78 (55.3%)	0.89
Hospitalization within 1 year	105 (61.4%)	86 (57.7%)	82 (58.2%)	0.76
Antipseudomonal antibiotic therapy within 3 months	55 (32.2%)	47 (31.5%)	60 (42.6%)	0.09
Non-antipseudomonal antibiotic therapy within 3 months	82 (48%)	75 (50.3%)	76 (53.9%)	0.58
Laboratory findings, median (IQR)				
Mean blood glucose during first 24 h (mg/dL)	137 (113‒165)	145 (112‒173)	157 (125‒198)	< 0.01
Hyperglycemia	38 (22.2%)	39 (26.2%)	60 (42.6%)	< 0.01

*APACHE* Acute Physiology and Chronic Health Evaluation score, *ICU* intensive care unit, *IQR* interquartile range, *PA Pseudomonas aeruginosa*, *SOFA* Sequential Organ Failure Assessment score

### Clinical features and outcomes

The clinical features at the time point when the peak density of PA was detected are presented in Table 2. The number of ETA performed after ICU admission until peak density of PA was detected was 2 (range 1–2) in the low-density group, 2 (1–3) in the moderate-density group, and 2 (1–4) in the high-density group, thus increasing with increased PA density. The frequency of VA-LRTI was less than 50% in the low-PA group, whereas it was about 80% in the moderate- and high-PA group. VAT accounted for about 70‒80% of VA-LRTI in each group, and the frequency of VAP tended to be higher in the high-PA group (*P* < 0.01). SOFA scores did not differ significantly among the three groups, whereas the CPIS tended to increase as the PA density increased. Levels of systemic inflammation markers, including white blood cell (WBC) count and C-reactive protein (CRP), were also higher in the high-PA group than in the other groups (*P* < 0.01). The CRP level in the moderate-density group was lower than that in the other two groups. Chest X-rays showed that pulmonary infiltrate lesions were more diffuse in the high-PA group.

**Table 2:** Patients’ characteristics at time of peak density of *Pseudomonas aeruginosa* isolation from endotracheal aspirates

	Low-PA group (*n* = 171)	Moderate-PA group (*n* = 149)	High-PA group (*n* = 141)	*P* value
VA-LRTI				< 0.01
Non-VA-LRTI	95 (55.6%)	34 (22.8%)	25 (17.7%)	
VAT	60 (35.1%)	96 (64.4%)	81 (57.5%)	
VAP	16 (9.4%)	19 (12.8%)	35 (24.8%)	
Length of MV, median (IQR), days	5 (2‒13)	11 (5‒20)	10 (4‒24)	< 0.01
SOFA	7 (5‒11)	8 (5‒11)	8 (5‒12)	0.51
CPIS	3 (2‒5)	4 (3‒5)	5 (3‒6)	< 0.01
Chest X-ray findings				0.02
No infiltrate	84 (49.1%)	61 (40.9%)	44 (31.2%)	
Unilateral infiltrates	58 (33.9%)	65 (43.6%)	66 (46.8%)	
Bilateral infiltrates	29 (17%)	23 (15.4%)	31 (22%)	
Laboratory findings, median (IQR)				
WBC (/µL)	10,900 (7600‒13,300)	9800 (6450‒13,450)	12,110 (7950‒16,600)	< 0.01
CRP (mg/dL)	7.5 (3.3‒14.1)	5.7 (2.5‒9.7)	8.2 (2.9‒16)	< 0.01
Tracheal aspirates analysis				
Neutrophil counts				< 0.01
0 to 1 +	47 (27.5%)	23 (15.4%)	27 (19.2%)	
2 + to 3 +	74 (43.3%)	60 (40.3%)	42 (29.8%)	
4 +	50 (29.2%)	66 (44.3%)	72 (51.1%)	
Culture results				
Commensal colonizer	96 (56.1%)	68 (45.6%)	55 (39%)	< 0.01
*Candida* sp.	46 (26.9%)	46 (30.9%)	42 (29.8%)	0.72
*Staphylococcus aureus*	29 (17%)	20 (13.4%)	21 (14.9%)	0.67
*Stenotrophomonas maltophilia*	32 (18.7%)	20 (13.4%)	13 (9.2%)	0.054
*Klebsiella pneumoniae*	10 (5.6%)	13 (8.7%)	10 (7.1%)	0.61
*Clinical outcomes*				
VFDs at 28 days				
Median (IQR), days	24 (6‒27)	21 (0‒27)	1 (0‒24)	< 0.01
Adjusted coefficient B (95% CI)^a^	Reference	1.1 (− 0.2 to 2.42)	− 1.94 (− 3.28 to − 0.61)	0.1^b^, < 0.01^c^
ICU mortality				
No. of cases	17 (9.9%)	22 (14.8%)	36 (25.3%)	< 0.01
Adjusted OR (95% CI)^a^	Reference	1.27 (0.46‒3.54)	2.78 (1.02‒7.58)	0.64^b^, 0.047^c^

*CI* confidence interval, *CPIS* Clinical Pulmonary Infection Score, *CRP* C-reactive protein, *ICU* intensive care unit, *IQR* interquartile range, *MV* mechanical ventilation, *OR* odds ratio, *PA Pseudomonas aeruginosa*, *SOFA* Sequential Organ Failure Assessment score, *VA-LRTI* ventilator-associated lower respiratory tract infection, *VAP* ventilator-associated pneumonia, *VAT* ventilator-associated tracheobronchitis, *VFDs* ventilator-free days, *WBC* white blood cell

^a^Adjusted for age, sex, respiratory diseases, APACHE II score at ICU admission, presence of VAT or VAP, length of MV, and SOFA score when the maximum density of PA was detected

^b^Moderate PA density versus low PA density

^c^High PA density versus low PA density

In the ETA analysis, the number of neutrophil cells increased as the PA density increased (*P* < 0.01). Regarding pathogenic bacteria besides PA, counts of *Stenotrophomonas maltophilia* were slightly lower in the high-PA group. VFDs at 28 days and ICU mortality were worse in the higher density groups.

After adjustment by multivariate analysis, the high-PA group still had significantly worse clinical outcomes than the low-PA group (VFDs, adjusted coefficient B − 1.94, 95% confidence interval [CI] − 3.28 to − 0.61, *P* < 0.01; ICU mortality, odds ratio [OR] 2.78, 95% CI 1.02‒7.58, *P* = 0.047), whereas the moderate-PA group did not differ significantly from the low-PA group.

### Risk factors for a high density of P. aeruginosa in the airway

Table 3 presents risk factors associated with high PA in univariate and multivariate analyses. Univariate logistic regression analysis revealed that longer duration (> 28 days) of MV, hyperglycemia, and use of non-antipseudomonal cephalosporins during the ICU stay were all significantly associated with high PA in MV patients. Patients with commensal colonizers during their ICU stay had a lower risk of having high PA. The main microorganisms among commensal colonizers were *Candida* spp. (34.1%), α-*Streptococcus* spp. (29.7%), and coagulase-negative staphylococci (14.3%). Marginal associations were observed for respiratory disease, low serum albumin levels at ICU admission, and use of antifungal antibiotics during the ICU stay.

**Table 3:** Risk factors for high-density *Pseudomonas aeruginosa* isolation during ICU stay

	Univariate	*P* value	Multivariate	*P* value
	OR (95% CI)		OR (95% CI)	
*Characteristics at ICU admission*				
Age	1.0 (0.99‒1.01)	0.79		NS
Female sex	1.22 (0.81‒1.84)	0.35		NS
Respiratory diseases	1.57 (0.96‒2.54)	0.069	1.9 (1.12‒3.23)	0.02
Albumin (g/dL)	0.79 (0.57‒1.09)	0.15		NS
APACHE II	1.01 (0.98‒1.03)	0.58		NS
*Events prior to peak density of PA isolation*				
Length of mechanical ventilation				
≤ 7 days	Reference			
8‒14 days	1.23 (0.73‒2.07)	0.44		NS
15‒21 days	0.75 (0.37‒1.53)	0.43		NS
22‒28 days	1.23 (0.55‒2.74)	0.61		NS
> 28 days	3.16 (1.7‒5.9)	< 0.01	3.07 (1.35‒6.97)	< 0.01
Hyperglycemia	1.9 (1.27‒2.84)	< 0.01	2.01 (1.26‒3.22)	< 0.01
Bacteria isolated in tracheal aspirates				
Commensal colonizer	0.52 (0.32‒0.84)	< 0.01	0.43 (0.26‒0.73)	< 0.01
*Stenotrophomonas maltophilia*	1.56 (0.76‒3.18)	0.22		NS
Antibiotic therapy				
Non-antipseudomonal cephalosporins	1.78 (1.16‒2.72)	< 0.01	2.17 (1.35‒3.49)	< 0.01
Antipseudomonal antibiotics	1.11 (0.74‒1.65)	0.61		NS
Antifungal antibiotics	1.51 (0.95‒2.39)	0.08		NS

*APACHE* Acute Physiology and Chronic Health Evaluation score, *CI* confidence interval, *ICU* intensive care unit, *NS* not significant, *OR* odds ratio, *PA Pseudomonas aeruginosa*

Multivariate logistic regression analysis confirmed that independent risk factors for high PA were longer duration (> 28 days) of MV (OR 3.07, 95% CI 1.35‒6.97, *P* < 0.01), use of non-antipseudomonal cephalosporins (OR 2.17, 95% CI 1.35‒3.49, *P* < 0.01), hyperglycemia (OR 2.01, 95% CI 1.26‒3.22, *P* < 0.01) during ICU stay, and respiratory diseases (OR 1.9, 95% CI 1.12‒3.23, *P* = 0.018). Isolation of commensal colonizers was independently associated with a lower risk of high PA (OR 0.43, 95% CI 0.26‒0.73, *P* < 0.01).

### Antibiotic therapy and outcomes

**Fig. 2:**
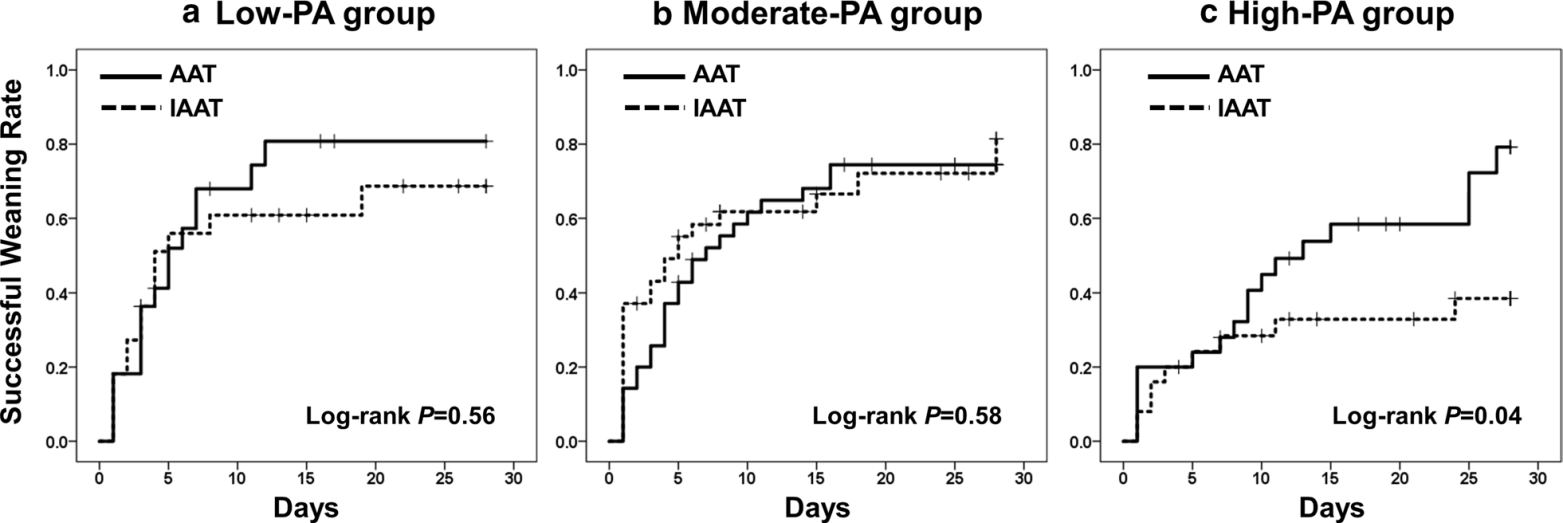
Kaplan‒Meier curves in propensity-matched patients for successful weaning from mechanical ventilation after the peak density of *Pseudomonas aeruginosa* was isolated. *AAT* appropriate antibiotic therapy, *IAAT* inappropriate antibiotic therapy, *PA Pseudomonas aeruginosa*

VFDs and ICU mortality in patients without VA-LRTI were similar in all groups, with median values of 20‒26 days and 4‒13%, respectively. Association of antibiotic therapy with outcomes in patients with VA-LRTI is shown in Table 4. In patients with VAT, VFDs in low- and moderate-PA groups did not vary significantly with appropriateness for antibiotic therapy. On the other hand, in the high-PA group, the number of VFDs in patients who received inappropriate antibiotic therapy (IAAT) was markedly lower than in those who received AAT. Patients with VAP had shorter VFDs, particularly in the moderate- and high-PA groups. ICU mortality tended to be decreased in VAP patients in the low- and moderate-PA groups who received AAT, as compared to those who did not (0‒16.7% vs. 45.5‒46.2%). Patients with high-PA VAP had a very high mortality rate, even with AAT (41.7%). We performed separate propensity score matching for VAT patients within each density group (Table 4, right panel). After matching, although not statistically significant due to the small numbers, AAT was also associated with an improvement in VFDs, but only in the high-PA group (median 0 vs. 17 days, *P* = 0.06), and not in the low- and moderate-PA groups. Kaplan–Meier analyses among matched patients showed that weaning from MV was almost identical between AAT and IAAT in both the low- and moderate-PA groups, while, in the high-PA group, a lower and later incidence of weaning success was observed with IAAT as compared to with AAT (Fig. 2).

**Table 4:** Association of antibiotic therapy with outcomes in patients with VA-LRTI

VAT	Before propensity scores matching			After propensity scores matching		
	IAAT	AAT	*P* value	IAAT	AAT	*P* value
Low PA						
No. of patients	33	26		22	22	
VFDs at 28 days, median (IQR)	24 (0–27)	22 (14–25)	0.37	24 (0–26)	23 (12–25)	0.85
ICU mortality	1 (3%)	3 (11.5%)	0.2	1 (4.6%)	3 (13.6%)	0.61
Moderate PA						
No. of patients	49	47		35	35	
VFDs at 28 days, median (IQR)	22 (0–27)	21 (0–25)	0.44	23 (0–27)	21 (0–25)	0.38
ICU mortality	3 (6.1%)	9 (19.2%)	0.054	2 (5.7%)	6 (17.1%)	0.26
High PA						
No. of patients	33	48		25	25	
VFDs at 28 days, median (IQR)	0 (0–20)	19 (0–24)	0.02	0 (0–22)	17 (0–22)	0.06
ICU mortality	9 (27.3%)	8 (16.7%)	0.25	9 (36%)	6 (24%)	0.54

VAP	IAAT	AAT	*P* value			
Low PA						
No. of patients	11	5				
VFDs at 28 days, median (IQR)	13 (0–22)	9 (5–19)	1			
ICU mortality	5 (45.5%)	0	0.07			
Moderate PA						
No. of patients	13	6				
VFDs at 28 days, median (IQR)	0 (0–19)	0 (0–23)	0.8			
ICU mortality	6 (46.2%)	1 (16.7%)	0.22			
High PA						
No. of patients	23	12				
VFDs at 28 days, median (IQR)	0 (0–0)	0 (0–13)	0.41			
ICU mortality	11 (47.8%)	5 (41.7%)	0.73			

*AAT* appropriate antibiotic therapy, *IAAT* inappropriate antibiotic therapy, *ICU* intensive care unit, *IQR* interquartile range, *PA Pseudomonas aeruginosa*, *VFDs* ventilator-free days, *VA-LRTI* ventilator-associated lower respiratory tract infection, *VAP* ventilator-associated pneumonia, *VAT* ventilator-associated tracheobronchitis

## Discussion

In this study, we investigated the relationship between airway PA density and clinical outcomes in MV patients, as well as the effects of antibiotic treatment. To the best of our knowledge, no previous study has highlighted the importance of airway PA density in MV patients or identified the risk factors for high PA. We found that high-PA was associated with worse clinical outcomes. Prolonged MV, non-antipseudomonal cephalosporins, hyperglycemia, and respiratory diseases were associated with higher risk, whereas the presence of commensal colonizers was associated with a lower risk for high-PA. Moreover, AAT for VAT patients was related to improved weaning from MV, but only in the high-PA group.

The relationship between airway PA density and clinical course has been reported for various respiratory diseases. A previous analysis of 385 patients with bronchiectasis showed that bacterial density was directly correlated with airway and systemic inflammatory markers, such as myeloperoxidase activity, neutrophil elastase activity, and tumor necrosis factor-α levels [30]. Airway bacterial density was also associated with exacerbation frequency and health-related quality of life. We found that MV patients with high-PA had more severe airway (neutrophils in the ETA) and systemic inflammation. CRP level was lower in the moderate-density group than in the other groups, which may be due to underlying hepatobiliary diseases that were slightly more frequent in the moderate-density group (26.9% in the moderate group vs. 19.9% in the other groups), affecting CRP production. The frequency of VA-LRTI was low in the low-PA group, while the high-PA group had more cases of VAP than other groups. Patients with high-PA may develop more severe respiratory infections, reflecting the higher inflammatory response in cases with high bacterial density. Our study found that the high-PA group had prolonged weaning from MV and higher ICU mortality. These results were similar when adjusted for the severity scores by multivariate analysis. Thus, the pathogenicity of PA, particularly when present at a high density in the airway, is expected to have a significant impact on the inflammatory response and the clinical course in MV patients. On the other hand, a recent study did not detect an association between bacterial density and progression to VAP in critically injured ventilated patients [31]. However, comparisons by pathogen species were not made in their study. The host immune response varies greatly depending on the type of pathogen [32–34], and the relationship between bacterial density and clinical outcomes in MV patients seen in our study may be different for pathogens other than PA. The relationship between colonization and progression to infection is not solely determined by bacterial density; the host immune status is also an important factor. Currently, potential markers such as volatile organic compounds [35], soluble triggering receptor expressed on myeloid cells 1 [36], and pentraxin 3 [37] are expected to be used for early diagnosis of VAP. Further research based on a comprehensive evaluation that includes these factors, as well as the bacterial burden, is needed to better understand respiratory infections in MV patients and to further optimize antibiotic use.

PA was isolated from ETA in 18% of ICU patients for surveillance and clinical cultures at our institution during the study period. The isolation rate of PA from ETA in previous reports varied from 5.8 to 27.9% [38, 39], and the decision which of these patients should be treated with antibiotics is an important issue in daily practice, particularly for patients with VAT, as an intermediate stage between airway colonization and VAP. The first randomized controlled trial of treatment for VAT demonstrated that patients who received AAT had higher numbers of VFD (median 12 vs. 2 days) and lower ICU mortality (18% vs. 47%) than the no antibiotic group [40]. Another randomized controlled trial showed that inhaled antibiotics in patients with VAT reduced symptoms of respiratory infections and increased weaning [41], and recent multicenter observational studies reported that appropriate antibiotic therapy reduced the progression of VAT to VAP [42, 43]. On the other hand, several studies reported no significant benefit of AAT [44, 45]. One of the problems with VA-LRTI studies is that although the diagnostic criteria for VAT and VAP are almost similar in each study, the interpretation of chest X-ray in the ICU may be affected by various artifacts and diseases other than pneumonia, sometimes making it difficult in practice to differentiate between VAT and VAP [46]. Other problems include differences in causative bacteria and antibiotic regimens used across studies. A recent guideline does not recommend routine use of antibiotics for VAT, given the inconsistent evidence of clinical benefit and the problem of adverse drug events [47]. The guideline suggests assessing the condition of individual patients and considering antibiotic therapy depending on disease severity. However, it is not yet known in which subgroups antibiotic therapy is particularly effective for VAT. In the present study, the number of airway neutrophils in VAT patients with high-PA was higher than that in other groups, suggesting that antibiotics may be particularly effective in these patients. Indeed, in our propensity score-matched analysis, AAT improved MV weaning outcomes only in VAT patients with high-PA. This relationship between PA density and the therapeutic effect of antibiotics was also supported by a recent study of patients with bronchiectasis [48].

We also explored the risk factors associated with high-PA and identified five significant variables: respiratory diseases at ICU admission, prolonged MV (> 28 days), non-antipseudomonal cephalosporins, hyperglycemia, and commensal colonizers during ICU stay. Respiratory diseases, duration of MV, and ineffective antibiotics against PA are well-known risk factors for PA colonization and infection in MV patients [23–25]. We newly identified hyperglycemia as an important risk factor for high-PA. In vitro and animal experiments showed that, as the blood glucose increased, the glucose concentration in the airway surface liquid also increased, which promotes the growth of airway PA and causes severe pneumonia [49, 50]. In human trials, airway glucose concentration was affected by blood glucose level and the presence of respiratory disease. Critically ill patients receiving MV showed high airway glucose levels [51]. For these reasons, the airways of MV patients with hyperglycemia may provide a more favorable environment for PA growth. We also found that isolation of commensal colonizers during the ICU stay was associated with a lower risk of high-PA. Bacteria have complex effects on each other. For example, *Candida* spp. downregulate quorum sensing of PA by farnesol [52], whereas oral commensal *Streptococcus* spp. suppress the growth of PA by producing hydrogen peroxide [53]. Thus, commensal colonizers may have protective effects against the growth of PA.

Our study had several limitations. First, as it was a retrospective analysis from a single ICU, our findings are subject to bias and may not be generalizable to all ICU patients. Second, 27.1% of our patients had been exposed to anti-pseudomonal antibiotics at the time of collecting the ETA sample, which may have affected the density of the isolated PA. However, the proportion of patients receiving anti-pseudomonal antibiotics was comparable among the three groups. Similarly, 29% of the samples in which the peak density of PA was detected had pathogens other than PA. Although the number of pathogens with a high bacterial density of 10^5^ cfu/mL or more was small in all cases (< 7.4%), bacteria have a complex relationship with each other, and these effects could not be completely ruled out in this study. Moreover, we could not evaluate respiratory infections caused by bacteria other than PA. Since these affect the length of MV, they may also have some effect on the peak density of PA. Third, we were unable to evaluate antibiotic therapy in detail, including the regimens and duration of administration, due to the small number of patients. The major limitation of this study was the long study period. Our clinical practices in ICU changed during the study period. For example, a VAP prevention bundle involving a semi-recumbent position, as well as the daily assessment of sedation and extubation readiness, was initiated in 2010. With the revision of the guidelines, therapeutic policies regarding antibiotic regimens and duration of VAP treatment were also changed, and optional treatments such as adjunctive macrolide use and inhaled antibiotics were adopted. Microbial changes in ICU were also observed, the prevalence of MRSA decreased, that of *Haemophilus* spp. increased, and that of PA did not change during this study period. The ratio of patients selected from the first half of the study period to those selected from the second half was similar in each PA density group; however, we were not able to assess the impact of these changes in clinical practice on the results. This is an important limitation of this study, and further research is required to understand the effects of bacterial density in MV patients.

## Conclusion

Airway PA density was associated with the clinical course and therapeutic efficacy of antibiotics used for patients on MV. The strict control of risk factors for high-PA identified in this study and the use of targeted antibiotic therapy for patients with VAT, particularly when high densities of PA are detected, may be useful to prevent ineffective antibiotic therapy and reduce antibiotic use. Further randomized controlled studies comparing aggressive risk factor modification with usual care, or antibiotics with placebo are required to confirm these findings.

## Data Availability

The data that support the findings of this study are available from the corresponding author upon reasonable request.

## Abbreviations

AAT: Appropriate antibiotic therapy
APACHE: Acute Physiology and Chronic Health Evaluation
cfu: Colony-forming units
CPIS: Clinical Pulmonary Infection Score
CI: Confidence interval
CRP: C-reactive protein
ETA: Endotracheal aspirates
High PA: High *Pseudomonas aeruginosa* density
HPF: High-power field
IAAT: Inappropriate antibiotic therapy
ICU: Intensive care unit
Low PA: low *Pseudomonas aeruginosa* density
Moderate PA: Moderate *Pseudomonas aeruginosa* density
MV: Mechanical ventilation
OR: Odds ratio
PA: *Pseudomonas aeruginosa*
SOFA: Sequential Organ Failure Assessment
VA-LRTI: Ventilator-associated lower respiratory tract infection
VAP: Ventilator-associated pneumonia
VAT: Ventilator-associated tracheobronchitis
VFDs: Ventilator-free days
WBC: White blood cell
